# Steeper structure-function relationship in eyes with than without a parapapillary deep-layer microvasculature dropout

**DOI:** 10.1038/s41598-018-32499-8

**Published:** 2018-09-21

**Authors:** Ji-Ah Kim, Eun Ji Lee, Hyunjoong Kim, Tae-Woo Kim

**Affiliations:** 1 Department of Ophthalmology, Seoul National University College of Medicine, Seoul National University Bundang Hospital, Seongnam, Korea; 2 Department of Applied Statistics, Yonsei University, Seoul, Republic of Korea

## Abstract

The degree of visual field (VF) loss can vary widely at a given level of retinal nerve fiber layer (RNFL) thickness. The cause of this variability is not fully understood. This cross-sectional study investigated whether the presence of choroidal microvasculature dropout (MvD) influences on the structure-function relationship among glaucomatous eyes. Seventy-one primary open-angle glaucoma (POAG) patients with choroidal MvD as determined by optical coherence tomography angiography (MvD+ group), and 71 age- and inferotemporal (IT) RNFL thickness-matched POAG patients without MvD (MvD– group) were included. VF sensitivity within the region corresponding to the IT RNFL sector was averaged using the total and pattern deviation fields. The slope of log-scale RNFL thickness versus VF defect was significantly steeper for the MvD+ than the MvD– group, as determined by both total and pattern deviation maps (*P* = 0.004 and <0.001, respectively). Both total and pattern VF deviation were significantly worse in the MvD+ than in the MvD– group (*P* = 0.002 and 0.007, respectively). Same results were obtained in subgroup analyses for eyes with thick and thin RNFL thickness (all *P* ≤ 0.027). These data suggest that parapapillary MvD is associated with poorer function of the remaining axons in eyes with POAG.

## Introduction

Glaucoma is characterized by a progressive loss of retinal ganglion cells (RGC) and their axons, accompanied by a reduction in visual field (VF) sensitivity. The disease is diagnosed and its severity assessed based on structural and functional evaluations. Therefore, understanding the relationship between structure and function is important. This is particularly because the clinical relationships between measures of structure and function differ markedly among individuals. For example, at a given level of retinal nerve fiber layer (RNFL) thickness, the degree of VF loss can vary widely. In clinical settings, this poses a diagnostic dilemma regarding the likelihood of disease and whether the severity of glaucoma should be primarily gauged on the basis of structure or function or a combination of both^1^.

One possible explanation for this variability may be the difference in the proportion of axons in the RNFL. The RNFL can be divided into two components: RGC axons and a residual component composed of glial cells and retinal vessels^2,3^. Inter-individual variations in the proportion of the two components^4,5^ may result in inter-individual differences in the number of axons included within a given thickness of the RNFL. In addition, media opacities and loss of RGC function before complete axonal atrophy may contribute to inter-individual variability in structure-function relationships^6^.

Recent studies^7–9^ demonstrated a parapapillary microvasculature dropout (MvD) in eyes with primary open angle glaucoma (POAG) using optical coherence tomography (OCT) angiography (OCTA). The parapapillary deep-layer MvD was associated with the thinner RNFL thickness and worse VF mean deviation^8^. Our group demonstrated that the parapapillary choroidal MvD defined by the OCTA corresponds to the true perfusion defect determined by indocyanine green angiography (ICGA)^10^. The parapapillary choroidal vasculature is downstream from the posterior ciliary artery^11–15^, which also perfuses prelaminar and laminar tissues^13–15^. Parapapillary choroidal MvD may be attributable to vascular compromise in the common arteriole before it divides into choroidal and optic nerve head (ONH) branches. If so, blood supply to the ONH may be insufficient in eyes with choroidal MvD. In addition, the prelaminar region is supplied by fine centripetal branches from the peripapillary choroid^16,17^. Because ICGA rarely detects choroidal vessels within the MvD^9^, the presence of parapapillary choroidal MvD may be considered a regional compromise of vessels supplying the ONH. Therefore, vascular supply to the deep ONH may be insufficient in eyes with choroidal MvD. Ischemia to the ONH affects the mitochondria^18^, which produce the high energy required for nerve conduction in the axons and for the maintenance of optimum neuronal function^19^. Therefore, hypoperfusion in the deep ONH tissues will reduce the function of the involved RGCs^20^. In addition, ischemia in the area of the MvD may disrupt the blood–optic nerve barrier, resulting in the release of vasoactive or toxic substances into the ONH^21^. These substances may also have a negative effect on the function of optic nerve axons.

Based on the concepts outlined above, we hypothesized that MvD may affect functional status at a given level of structural damage. Although it has been demonstrated that eyes with MvD had worse VF damage compared to eyes without MvD, the RNFL thickness were also thinner in eyes with MvD^8^. Therefore, it is unknown whether MvD affects the structure-function relationship. The purpose of the present study was to compare the structure-function relationship between POAG eyes with and without MvD, as determined by OCTA.

## Results

Initially, the study included 335 eyes with POAG from 335 patients. Of these, 13 were excluded because of poor quality angiography images. There was excellent interobserver agreement in the detection of MvD (κ = 0.930) and in measuring MvD extent [intraclass correlation coefficient (ICC) = 0.926], parapapillary atrophy (PPA) area (ICC = 0.964) and disc area (ICC = 0.977).

### Distribution of MvD

Of the 322 eyes of patients with POAG, 193 (59.9%) had parapapillary MvD. Most (n = 183, 94.8%) MvDs were located in the inferotemporal (IT) sector, with six (3.1%) located in the superotemporal, two (1.0%) in the temporal, and two (1.0%) in the inferonasal sector.

### Patient Characteristics and MvD

**Figure 1:**
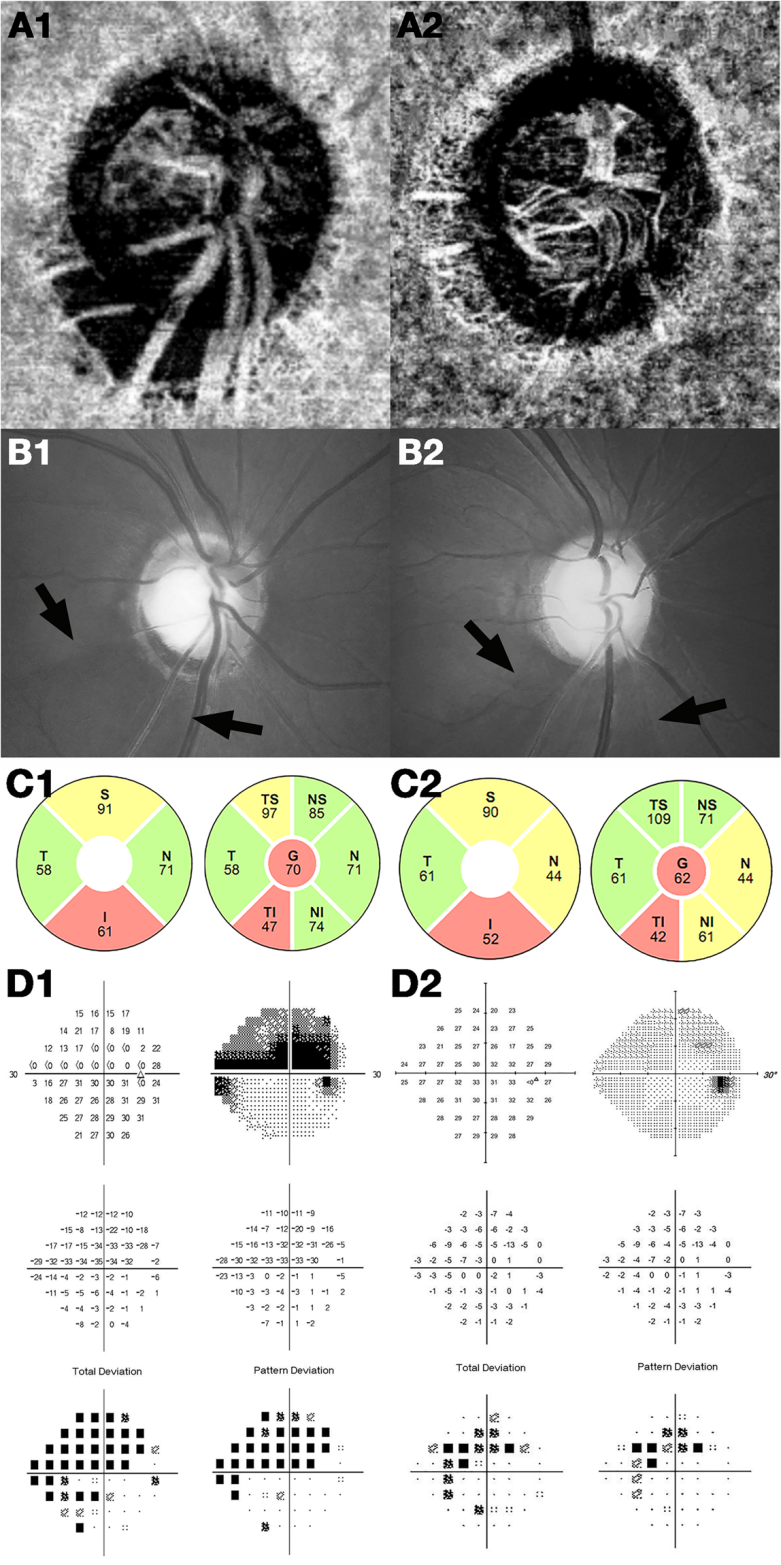
Optical coherence tomography angiography images of the parapapillary deep layer, including the retinal pigment epithelium, choroid, and anterior sclera (**A**), red-free fundus photographs (**B**), optical coherence tomography – retinal nerve fiber layer (RNFL) sector maps (**C**), and Humphrey visual field (HVF) maps (**D**) of glaucomatous eyes with (left column) and without (right column) choroidal microvasculature dropout (MvD). HVF results in the area of interest were worse in the eyes with (**D1**) than without (**D2**) MvD, although the inferotemporal RNFL was thicker in the former (**C1**) than the latter (**C2**).

The clinical and ocular characteristics of the RNFL thickness-matched POAG patients with and without MvD are shown in Table 1. The area of β zone PPA was significantly larger in the MvD+ group (*P* < 0.001), whereas the use of antihypertensive drugs was significantly higher in the MvD– group (*P* = 0.016). There were no significant between-group differences in age, gender, RNFL thickness, disc area, IOP, axial length, spherical equivalent, blood pressures (BPs), migraine, and self-reported cold extremities. Representative cases are shown in Fig. 1.

**Table 1:** Baseline Characteristics of the Participants.

Clinical characteristics	Eyes with Microvasculature Dropout, *n* = 71	Eyes without Microvasculature Dropout, *n* = 71	*P-*value
Age, y	58.08 ± 10.84	57.68 ± 10.35	0.819^*^
Sex, male/female	27/44	36/35	0.128^†^
IT RNFL thickness, μm	65.51 ± 15.60	65.14 ± 15.56	0.889^*^
Disc area, mm^2^	2.24 ± 0.33	2.31 ± 0.50	0.304^*^
IOP at OCTA, mmHg	12.37 ± 3.12	12.32 ± 2.54	0.918^*^
CCT, μm	545.17 ± 27.75	547.10 ± 35.13	0.717^*^
Axial length, mm	24.31 ± 1.21	23.99 ± 1.02	0.091^*^
Spherical equivalent, D	−1.30 ± 2.21	−1.11 ± 2.37	0.634^*^
Visual field MD, dB	−6.48 ± 3.86	−4.93 ± 3.72	**0.016** ^*^
βPPA area, mm^2^	0.83 ± 0.55	0.53 ± 0.37	**<0.001** ^*^
Systolic BP, mmHg	123.96 ± 14.16	124.28 ± 15.97	0.898^*^
Diastolic BP, mmHg	75.48 ± 10.13	73.42 ± 9.03	0.204^*^
Mean arterial pressure, mmHg	91.64 ± 10.67	90.38 ± 10.57	0.480^*^
Ocular perfusion pressure, mmHg	79.27 ± 10.28	78.06 ± 10.48	0.487^*^
Hypertension medication, %	19.7	38.0	**0.016** ^**†**^
Migraine, %	16.9	18.3	0.826^†^
Self-reported cold extremities, %	28.1	26.8	0.851^†^
Average of total deviation value corresponding to IT RNFL, dB	−7.66 ± 6.74	−4.89 ± 3.48	**0.002** ^*^
Average of pattern deviation value corresponding to IT RNFL, dB	−6.79 ± 6.57	−4.46 ± 2.99	**0.007** ^*^

Values with statistical significance are in boldface.

IT RNFL = inferotemporal retinal nerve fiber layer; IOP = intraocular pressure; OCTA = optical coherence tomography angiography; CCT = central corneal thickness; D = diopters; MD = mean deviation; PPA = parapapillary atrophy; BP = blood pressure; dB = decibel.

^*^Independent samples *t* test.

^†^Chi-square test.

### Comparison of Structure-Function Relationship between Groups

**Figure 2:**
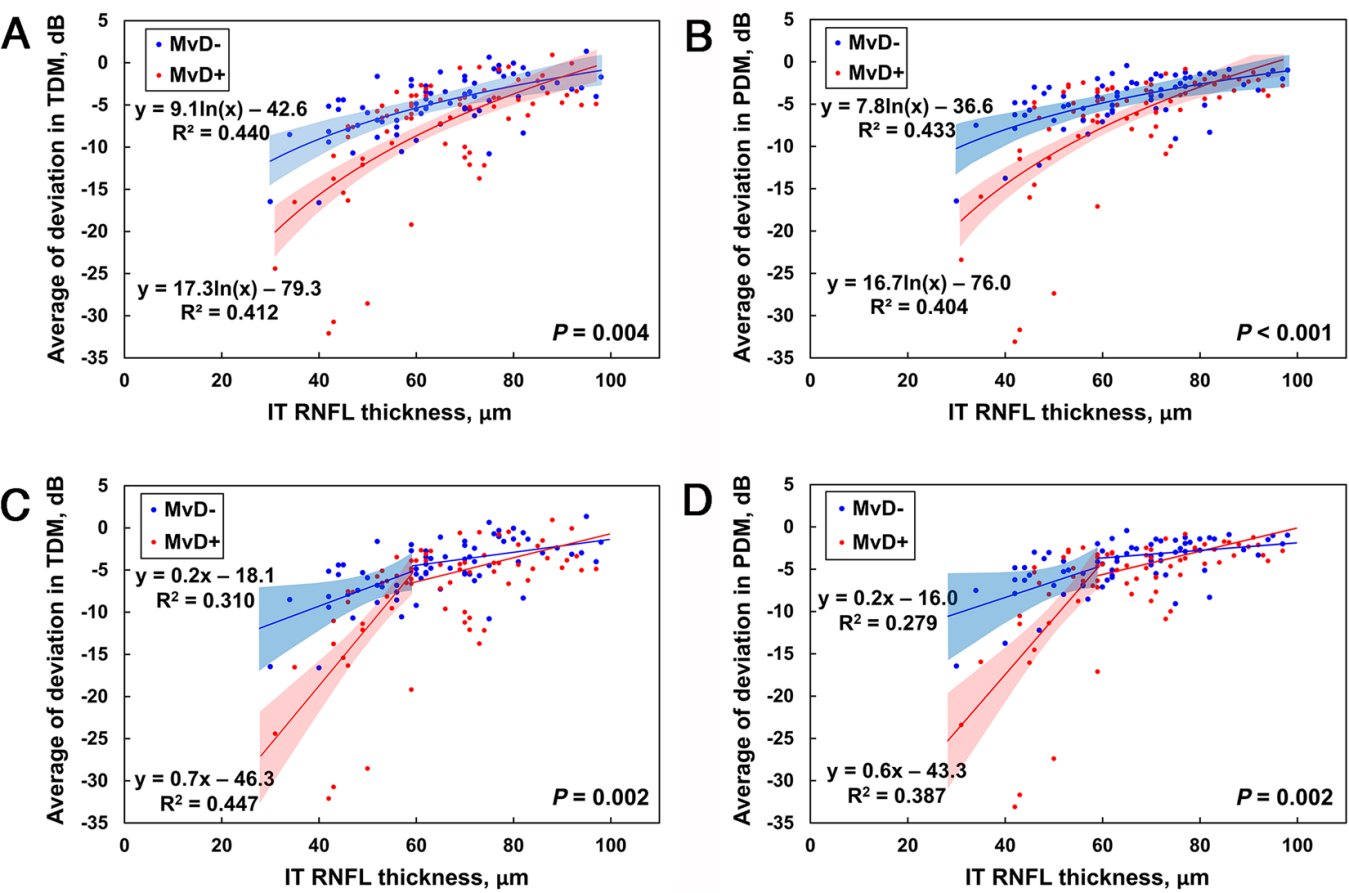
Scatterplots showing the relationship between IT RNFL thickness and corresponding total (**A**) and pattern (**B**) visual field deviation averages expressed in decibels by logarithmic regression analyses in eyes with and without MvD. The structure-function relationships were steeper in MvD+ (solid lines) than in MvD− (dotted lines) eyes on both the total and pattern deviation maps. (**C**,**D**) Analysis of the same scatterplots using the Hockey-stick model, again showing steeper slopes below the tipping point in MvD+ than in MvD− eyes on both the total and pattern deviation maps (both *P* = 0.002). (IT = inferotemporal, RNFL = retinal nerve fiber layer, MvD = microvasculature dropout, TDM = total deviation map, PDM = pattern deviation map, dB = decibel)

A significant correlation between IT RNFL thickness and VF defect at the corresponding area was found for both MvD+ and MvD− group at the total (R^2^ = 0.412, 0.440, respectively, both *P* < 0.001) and pattern deviation map (R^2^ = 0.404, 0.433, respectively, both *P* < 0.001). A univariate regression analysis showed that age, beta-zone PPA area, and MvD had a significant or marginal influence of *P* < 0.1 on the structure and function relationship between ln(RNFL thickness) and total deviation average (Table 2). Beta-zone PPA area and MvD had such influence (i.e., *P* < 0.1) on the relationship between ln(RNFL thickness) and pattern deviation average (Table 3). In the multivariate model, only MvD had a significant influence on the structure-function relationship between ln(RNFL thickness) and total and pattern deviation averages (*P* = 0.001 and 0.004, respectively). The structure-function relationship was significantly steeper in the MvD+ than in the MvD− group, on both total (y = 17.25 ln(x) − 79.30 vs. y = 9.09 ln(x) − 42.58, *P* = 0.004) and pattern (y = 16.67 ln(x) − 76.00 vs. y = 7.75 ln(x) − 36.61, *P* < 0.001) deviation maps (Fig. 2A,B). Significantly steeper structure-function relationship was also found in the MvD+ than in the MvD− group on both total and pattern deviation maps (*P* = 0.006 and 0.002, respectively) after accounting factors which had an influence (*P* < 0.1).

**Table 2:** Factors Associated with the Slope of Structure-Function Relationship in Total Deviation Map Corresponding to the IT RNFL sector.

Variables	Univariate			Multivariate		
	β	95% CI	*P*-value	β	95% CI	*P*-value
Age, *per 1 yr older*	−0.062	−0.132, 0.009	0.085	−0.061	−0.128, 0.006	0.073
Female gender	0.039	−1.467, 1.545	0.959			
CCT, *per 1 µm larger*	0.008	−0.015, 0.032	0.480			
AXL, *per 1 mm larger*	0.089	−0.586, 0.765	0.795			
SE, *per 1 D larger*	0.076	−0.256, 0.408	0.651			
Disc area, *per 1 mm*^2^ *larger*	−0.002	−1.762, 1.758	0.998			
βPPA area, *per 1 mm*^2^ *increase*	−1.885	−3.374, −0.395	**0.014**	−1.126	−2.626, 0.374	0.140
Baseline IOP, *per 1 mmHg higher*	−0.187	−0.448, 0.075	0.161			
MvD	2.852	1.446, 4.258	**<0.001**	2.488	1.026, 3.951	**0.001**
SBP, *per 1 mmHg higher*	0.003	−0.047, 0.052	0.920			
DBP, *per 1 mmHg higher*	−0.028	−0.105, 0.050	0.483			
MAP, *per 1 mmHg higher*	−0.013	−0.084, 0.057	0.707			
OPP, *per 1 mmHg higher*	0.000	−0.072, 0.072	0.998			
Cold extremities	−1.148	−2.800, 0.505	0.172			
Migraine	0.554	−1.399, 2.506	0.576			
Antihypertensive medication	0.028	−1.614, 1.670	0.973			

^*^Only variables with *P* < 0.1 on univariate analysis were included in the multivariate model.

Values with statistical significance are in boldface.

IT = inferotemporal; RNFL = retinal nerve fiber layer; CCT = central corneal thickness; AXL = axial length; SE = spherical equivalent; βPPA = β-zone parapapillary atrophy; IOP = intraocular pressure; MvD = microvasculature dropout; SBP = systolic blood pressure; DBP = diastolic blood pressure; MAP = mean arterial pressure; OPP = mean ocular perfusion pressure.

**Table 3:** Factors Associated with the Slope of Structure-Function Relationship in Pattern Deviation Map Corresponding to the IT RNFL sector.

Variables	Univariate			Multivariate		
	β	95% CI	*P*-value	β	95% CI	*P*-value
Age, *per 1 yr older*	−0.020	−0.088, 0.048	0.565			
Female gender	0.470	−0.966, 1.906	0.519			
CCT, *per 1 µm larger*	0.011	−0.011, 0.034	0.334			
AXL, *per 1 mm larger*	−0.099	−0.744, 0.546	0.762			
SE, *per 1 D larger*	0.153	−0.164, 0.469	0.342			
Disc area, *per 1 mm*^2^ *larger*	0.238	−1.442, 1.919	0.779			
βPPA area, *per 1 mm*^2^ *increase*	−1.642	−3.070, −0.213	**0.025**	−0.969	−2.433, 0.495	0.193
Baseline IOP, *per 1 mmHg higher*	−0.057	−0.308, 0.195	0.657			
MvD	2.405	1.045, 3.765	**0.001**	2.112	0.685, 3.539	**0.004**
SBP, *per 1 mmHg higher*	0.004	−0.044, 0.051	0.881			
DBP, *per 1 mmHg higher*	−0.027	−0.101, 0.047	0.473			
MAP, *per 1 mmHg higher*	−0.012	−0.080, 0.055	0.717			
OPP, *per 1 mmHg higher*	−0.009	−0.077, 0.060	0.803			
Cold extremities	−1.252	−2.827, 0.323	0.118			
Migraine	0.291	−1.575, 2.158	0.758			
Antihypertensive medication	0.522	−1.044, 2.088	0.511			

^*^Only variables with *P* < 0.1 on univariate analysis were included in the multivariate model.

Values with statistical significance are in boldface.

IT = inferotemporal; RNFL = retinal nerve fiber layer; CCT = central corneal thickness; AXL = axial length; SE = spherical equivalent; βPPA = β-zone parapapillary atrophy; IOP = intraocular pressure; MvD = microvasculature dropout; SBP = systolic blood pressure; DBP = diastolic blood pressure; MAP = mean arterial pressure; OPP = mean ocular perfusion pressure.

Using the ‘Hockey-Stick’ model, a tipping point was observed for both the total (62.50 µm) and pattern (62.50 µm) deviation maps. The MvD+ group showed a steeper slope below the tipping point on both the total (*P* = 0.002) and pattern (*P* = 0.002) deviation maps (Fig. 2C,D). However, there were no between-group differences in slopes above the tipping point on both maps.

### Comparison of Visual Field Deviation between Groups

VF total deviation (−7.66 dB vs. −4.89 dB, *P* = 0.002) and VF pattern deviation (−6.79 dB vs. −4.46 dB, *P* = 0.007) were significantly worse in the MvD+ than in the MvD− group. The comparison of VF deviation was also performed in subgroups with RNFL thickness greater (Thick RNFL group) and smaller (Thin RNFL group) than the above mentioned tipping point (i.e., 62.50 µm). Thirty-eight eyes and 33 eyes were included in the Thick and Thin RNFL group, respectively in each MvD+ and MvD− group. VF sensitivity of each group is shown in Table 4. Total and pattern deviation scores were significantly poorer in MvD+ than MvD− eyes both in the Thick and Thin RNFL group (all *P* ≤ 0.027).

**Table 4:** Comparison of Visual Field Deviation between Microvasculature Dropout Positive and Negative Eyes for Subgroups Divided by IT RNFL Thickness.

Groups divided by IT RNFL thickness	Deviation map type	Deviation value		*P*-value
		MvD+	MvD−	
Thin group (<62.5 μm), *n* = 66	Total	−10.70 ± 8.14	−6.83 ± 3.42	**0.016**
	Pattern	−9.73 ± 8.42	−6.12 ± 3.13	**0.027**
Thick group (>62.5 μm), *n* = 76	Total	−5.02 ± 3.64	−3.20 ± 2.54	**0.013**
	Pattern	−4.23 ± 2.43	−2.98 ± 1.90	**0.015**

Values are shown in mean ± standard deviation.

Values with statistical significance are in boldface.

IT = inferotemporal; RNFL = retinal nerve fiber layer; MvD = microvasculature dropout.

### Relationship Between the Extent of MvD and RNFL Thickness

**Figure 3:**
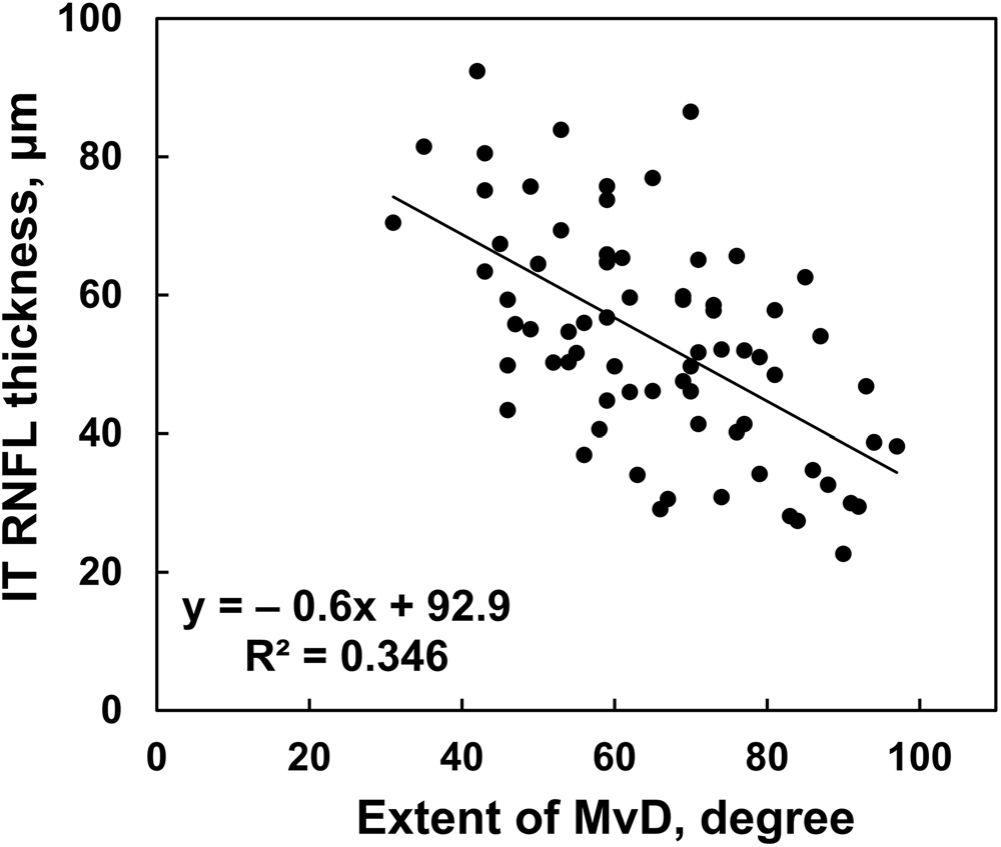
Scatterplot of optical coherence tomography inferotemporal (IT) retinal nerve fiber layer (RNFL) thickness versus the extent of choroidal microvasculature dropout (MvD). The fitted linear regression illustrates the inverse relationship between these variables.

There was a significant negative correlation between RNFL thickness and the extent of MvD (R^2^ = 0.346, *P* < 0.001, Fig. 3).

## Discussion

This study compared the structure-function relationship between POAG eyes with and without parapapillary MvD. Eyes with parapapillary MvD had a worse VF deviation and steeper structure-function relationship, with the between-group difference in average VF deviation increasing as IT RNFL thickness decreased. To our knowledge, no previous study has assessed the influence of parapapillary MvD on the structure-function relationship in eyes with glaucoma.

In the present study, the degree of VF loss was analyzed using both total and pattern deviation plots. The total deviation represents the deviation from expected values based on the age-matched normal database. However, the values measured in the total deviation plot may be influenced by media opacity. In contrast, pattern deviation plots use adjusted values to eliminate “background noise”. But this general correction may not always be appropriate. Removing all diffuse VF loss in pattern deviation values may underestimate glaucomatous VF damage in some patients with advanced VF damage^22^. Therefore we analyzed both plots.

Although low diastolic BP was reported to be one of the factors associated with parapapillary MvD in glaucomatous eyes^8^, the present study found that diastolic BP did not differ significantly in the two groups. This discrepancy may have been due to differences in the percentages of patients in the two studies who were taking oral systemic antihypertensive drugs.

Several hypotheses may explain why VF defect at the same level of RNFL is worse in eyes with than without MvD. First, the parapapillary choroidal vasculature is downstream from the posterior ciliary artery^11–15^, which also perfuses the prelaminar and laminar tissues^13–15^. Therefore, the presence of MvD may suggest a vascular insufficiency in the deep ONH structure. Nerve conduction is an energetically costly process^19,23^. Therefore, hypoperfusion of the prelaminar and/or laminar region may reduce nerve conduction, worsening VF sensitivity. Second, ischemia in the area of the MvD may promote the disruption of the blood–optic nerve barrier^21^, which is known to occur in glaucomatous optic nerve head^24,25^. Disruption of the blood-optic nerve barrier potentially could result in the release of vasoactive or toxic substances into the ONH. Those substances may also have a negative effect on the function of optic nerve axons.

The slope of the structure-function relationship was steeper in the MvD+ than in the MvD– group, indicating that the between-group difference in VF sensitivity tended to be greater in eyes with thinner than thicker RNFL. This may be attributed to the negative correlation between RNFL thickness and the extent of MvD. A larger MvD may influence the function of a larger number of axons. Alternatively, it is possible this may be due to large variability of VF sensitivity independent of RNFL thickness in eyes with thin RNFL near the floor level. However, the worse VF deviation was also observed in eyes with thick RNFL. Therefore, it may be reasonably proposed that the larger intergroup (MvD+ vs. MvD−) difference in VF deviation in eyes with thinner RNFL is at least partly due to larger extent of MvD in these eyes.

The current study had several limitations. The first was its cross-sectional design. We compared the slopes of the structure-function relationship of the two groups. In a longitudinal study, the natural characteristics of MvD would have been more apparent. However, OCTA was commercialized relatively recently, and longitudinal studies of glaucoma usually take years. Our study provided meaningful results and can indicate the direction of future research. Second, precise evaluation of the MvD may have been hampered by flow projection artifacts caused by the moving shadows cast by flowing blood cells^26,27^. These possibilities should be considered when evaluating OCTA images in future studies. Third, there is possibility that eyes with larger PPA may have more visible MvD. Further study is needed to clarify the relationship between the PPA and MvD.

In conclusion, at the same level of peripapillary IT RNFL thickness, eyes with MvD had poorer VF test results and steeper structure-function relationships than eyes without MvD. This finding indicates that parapapillary MvD negatively affects the function of the remaining axons in eyes with POAG.

## Methods

### Participants

This study involved POAG patients who were enrolled in the Investigating Glaucoma Progression Study (IGPS), an ongoing prospective study of glaucoma patients at the Glaucoma Clinic of Seoul National University Bundang Hospital, and with OCTA results. All subjects provided written informed consent. The study protocol was approved by the Institutional Review Board of Seoul National University Bundang Hospital and followed the tenets of the Declaration of Helsinki.

All participants underwent comprehensive ophthalmic examinations, which included assessments of best-corrected visual acuity (BCVA), Goldmann applanation tonometry, a refraction test, slit-lamp biomicroscopy, gonioscopy, stereo disc photography, red-free fundus photography (EOS D60 digital camera, Canon, Utsunomiyashi, Tochigiken, Japan), central corneal thickness (CCT) measurement (Orbscan II, Bausch & Lomb Surgical, Rochester, NY, USA), and axial length measurement (IOLMaster version 5, Carl Zeiss Meditec, Dublin, CA, USA). Peripapillary RNFL thickness was measured by spectral-domain OCT (SD-OCT; Spectralis, Heidelberg Engineering, Heidelberg, Germany). Other ophthalmic examinations included standard automated perimetry (SAP, Humphrey Field Analyzer II 750, 24-2 Swedish interactive threshold algorithm, Carl Zeiss Meditec) and OCTA (DRI OCT Triton; Topcon, Tokyo, Japan). Spectral-domain OCT, SAP, and OCTA were performed on the same day. Intraocular pressure (IOP) was measured at the time of OCTA. Clinical history was also taken from participants, including demographic characteristics and the presence of cold extremities and other systemic conditions. Systolic and diastolic BP were measured at the time of OCTA. BP was measured with an automatic sphygmomanometer in seated subjects after a 5-minute rest. Mean arterial pressure (MAP) was calculated using the expression MAP = diastolic BP + 1/3 (systolic BP − diastolic BP), and ocular perfusion pressure (OPP) was calculated using the equation OPP = MAP – IOP at the time of OCTA.

POAG was defined as the presence of an open iridocorneal angle, signs of glaucomatous optic nerve damage (i.e., neuroretinal rim thinning, notching, or a RNFL defect), and a glaucomatous VF defect. A glaucomatous VF defect was defined as a defect conforming with one or more of the following criteria: (1) outside normal limits on a glaucoma hemifield test, (2) three abnormal points with a <5% probability of being normal and one abnormal point with a <1% probability by pattern deviation, or (3) a pattern standard deviation of probability <5% confirmed on two consecutive reliable tests (fixation loss rate of ≤20% and false-positive and false-negative error rates of ≤25%).

The exclusion criteria were eyes with a BCVA worse than 20/40, a spherical equivalent of <−6.0 D or >+3.0 D, a cylinder correction of <−3.0 D or >+3.0 D, a history of intraocular surgery with the exception of uneventful cataract surgery or trabeculectomy, or retinal or neurological diseases. Eyes with a tilted (ratio of minimum to maximum optic disc diameter <0.75) or torted (angle of tilt >15°) disc were also excluded^28^. Eyes with advanced glaucoma (VF mean deviation worse than −15 decibels (dB)) were additionally excluded as RNFL thinning may reach horizontal asymptotes in advanced stage of disease^29,30^. Because MvD was found almost exclusively at the IT sector, the between-group structure-function relationships were compared only for the IT sector. Patients with MvD in the IT parapapillary area were assigned to the MvD+ group. Eyes with MvD at other sectors were excluded from the analysis. If a subject had IT MvD in only one eye, that eye was included in the analysis. If both eyes had IT MvD or neither eye had MvD, one eye was randomly selected for inclusion in the study.

To compare the slopes of the structure-function graphs in the MvD+ and MvD− group, patients in the two groups were matched 1:1 by IT RNFL thickness and age.

### Retinal Nerve Fiber Layer Thickness Measurement

RNFL thickness was measured using by spectral domain OCT (Spectralis, Heidelberg Engineering) circumpapillary RNFL circle scan, which has shown excellent reproducibility in measurements of peripapillary RNFL thickness^31,32^. Only scans with a quality score ≥20 were included. The Spectralis OCT software (version 6.0) allows for automatic segmentation of the upper and lower borders of the RNFL to calculate the RNFL thickness. This software divides peripapillary RNFL thicknesses into 4 quadrants, with the superior and inferior quadrants further divided into nasal and temporal sectors.

### OCTA

The optic nerve and peripapillary area were imaged using a commercially available OCTA device. Each image set consisted of two raster volumetric patterns (one with vertical and the other with horizontal priority), which covered a 4.5 mm × 4.5 mm area centered on the optic disc. The OCTA images were coregistered with OCT B-scans obtained concurrently, to enable simultaneous visualization of vascular signal and structure.

**Figure 4:**
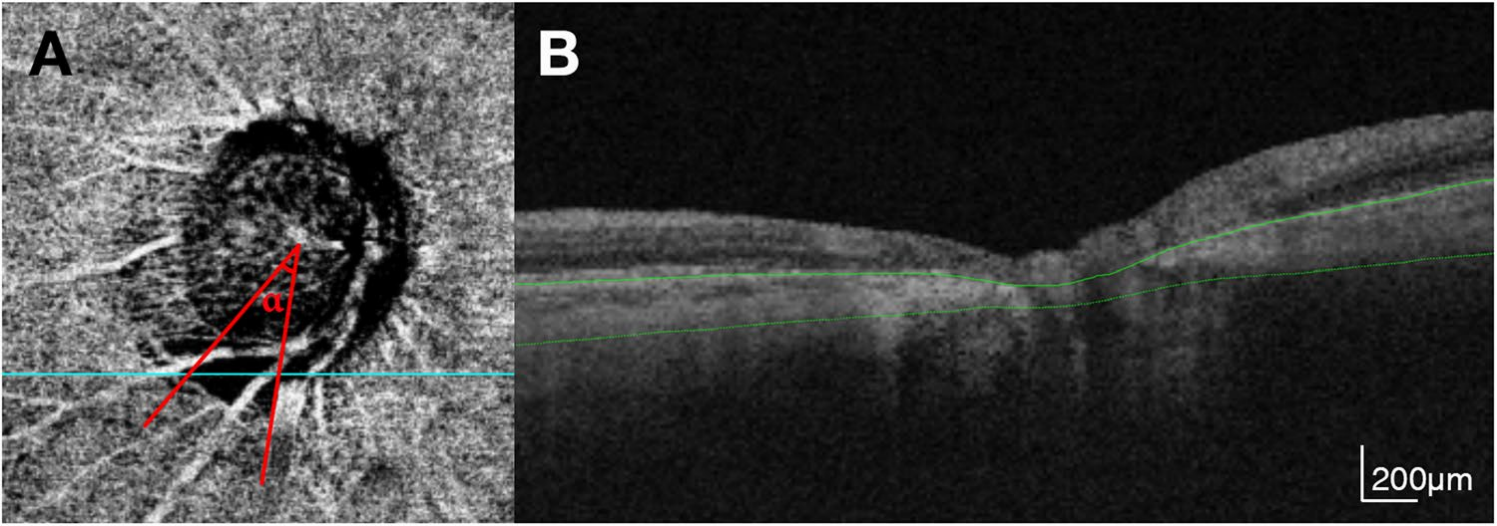
(**A**) En face optical coherence tomography angiography image for the choroidal layer. Angle α indicates the extent of choroidal microvasculature dropout. (**B**) B-scan image at the location marked with blue line in A. Green lines indicate the segmented layer.

The choroidal microvasculature in the peripapillary area was evaluated on the 4.5 × 4.5-mm choroid/disc vessel density maps of ONH OCTA images. These images contain layers below the retinal pigment epithelium, including the choroid and anterior sclera (Fig. 4B). MvD was defined as a focal sectoral capillary dropout without any visible microvascular network. Before defining the MvD, automated segmentation of the choroid by the device was checked, with false segmentations manually corrected. An apparent circumferential width of the area with capillary dropout more than twice that of the width of visible juxtapapillary microvessels was considered a disruption of the microvascular network and was defined as MvD^33^. The area covered by large retinal vessels was included as part of the MvD when the MvD extended beyond the vessels; if not, the area covered by the large vessels was not included in the MvD. The extent of MvD was measured as the angular extent of the MvD using Image J software [National Institute of Health, Bethesda, MD, USA (http://imagej.nih.gov/ij/)] (Fig. 4A). This measurement was made by first identifying the two points at which the borders of an MvD area met the clinical optic disc margin. Lines connecting the disc center and the two points were drawn, and these lines were used to measure the angular extent. Two independent observers (J.A.K. and E.J.L.) blinded to the clinical characteristics of the subjects identified MvDs and measured the extent of MvD at the IT parapapillary region. Disagreements between these two observers regarding the presence/absence of the MvD were resolved by a third adjudicator (T.W.K.). The extent of the MvD was defined as the mean measured by the two observers. Eyes were excluded from analysis when the quality of the OCTA images was poor (i.e., blurred images that hampered the delineation of the MvD).

### Analyses of Parapapillary Atrophy and Disc Area

The area of β-zone PPA which defined as the area without retinal pigment epithelium and with intact Bruch’s membrane and disc area were determined using the software provided by the Spectralis viewer (Heidelberg Eye Explorer software version 1.9.10.0; Heidelberg Engineering), as previously described^34–36^. Two experienced ophthalmologists (J.A.K. and E.J.L.) blinded to the clinical characteristics of the subjects measured PPA and disc area using the built-in caliper tool of the Spectralis OCT system. Each observer measured each PPA area and disc area, with the mean of the two measurements included in the analysis.

### Assessment of Structure-Function Relationship

**Figure 5:**
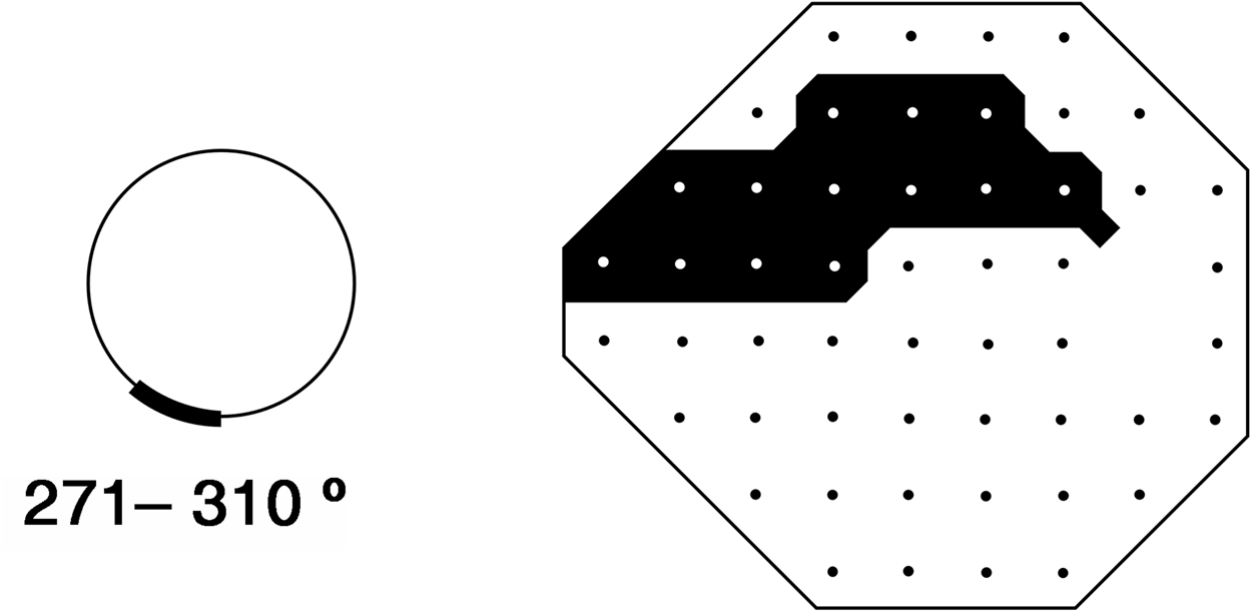
A schematic illustrating the superior arcuate field region corresponding to the inferior retinal nerve fiber layer arcuate sector from 271° to 310° according to the Garway-Heath map.

The structure-function relationship was evaluated only for the IT sector. The RNFL thickness of the IT sector (271–310°) was obtained from the printout of the SD-OCT measurement of peripapillary RNFL thickness. The VF region corresponding to the OCT IT sector was determined using Garway-Heath maps (Fig. 5)^37^, and the total and pattern deviations in this region were evaluated.

VF sensitivity is expressed conventionally on a dB (logarithmic) scale. To calculate the mean total and pattern deviation of the IT sector, the dB levels in the location of the total and pattern deviation fields were converted to a linear scale using the equation dB = 10 log_10_ (1/*L*) before averaging the data within each sector. The averaged data were then converted back to dB units^38,39^.

Because log(RNFL thickness) and VF sensitivity have a linear relationship, a logarithmic (y = a*ln(x) + b) regression model was fitted, as described previously^38,40,41^. The interaction term between RNFL thickness and group was included, allowing evaluation of the difference in slopes between the two groups. Factors influencing the structure-function relationship were identified by regression analysis. The slopes for the structure and function were compared after accounting for the factors which had an influence of *P* < 0.1 on the structure-function relationship in the univariate regression analysis.

### Data analysis

Independent sample *t*-tests, chi-square test were used to compare the group differences as applicable. Interobserver agreement on the presence or absence of MvD was assessed using kappa statistics (κ value), and interobserver agreement on the extent of MvD and the area of PPA and disc area were assessed by calculating ICCs. Logarithmic regression for the interaction between RNFL thickness and group was modeled as y = a*ln(x) + b*I*ln(x) + c + d*I, where I = 1 if MvD+, = 0 if MvD−. The coefficients a and c indicate the slope and intercept of MvD− group. The coefficients b and d refer to the difference in slopes and intercepts between the two groups. For the ‘Hockey-Stick model’, a tipping point was calculated using Davies’ test. All statistical analyses were performed using SPSS software (version 19.0, SPSS, Chicago, IL, USA) and R statistical packages version 3.3.3 (available at http://www.R-project.org; accessed on July 9, 2017) with *P* < 0.05 defined as statistically significant. The data are presented as mean ± SD values except where stated otherwise.

## Data Availability

Data supporting the findings of the current study are available from the corresponding author on reasonable request.
